# Differentially expressed genes during spontaneous lytic switch of Marek's disease virus in lymphoblastoid cell lines determined by global gene expression profiling

**DOI:** 10.1099/jgv.0.000744

**Published:** 2017-05-05

**Authors:** William N. Mwangi, Deepali Vasoya, Lydia B. Kgosana, Mick Watson, Venugopal Nair

**Affiliations:** ^1^​ Avian Viral Diseases Programme, UK-China Centre of Excellence on Avian Disease Research, The Pirbright Institute, Pirbright, Surrey, UK; ^2^​ Division of Genetics and Genomics, The Roslin Institute, R(D)SVS, University of Edinburgh, Easter Bush, Midlothian, UK

**Keywords:** Marek’s disease virus, transformation, latency, reactivation

## Abstract

Marek’s disease virus (MDV), an alphaherpesvirus of poultry, causes Marek’s disease and is characterized by visceral CD4^+^TCRαβ^+^ T-cell lymphomas in susceptible hosts. Immortal cell lines harbouring the viral genome have been generated from *ex vivo* cultures of MD tumours. As readily available sources of large numbers of cells, MDV-transformed lymphoblastoid cell lines (LCLs) are extremely valuable for studies of virus–host interaction. While the viral genome in most cells is held in a latent state, minor populations of cells display spontaneous reactivation identifiable by the expression of lytic viral genes. Spontaneous reactivation in these cells presents an opportunity to investigate the biological processes involved in the virus reactivation. For detailed characterization of the molecular events associated with reactivation, we used two lymphoblastoid cell lines derived from lymphomas induced by pRB1B-UL47eGFP, a recombinant MDV engineered to express enhanced green fluorescent protein (EGFP) fused with the UL47. We used fluorescence-activated cell sorting to purify the low-frequency EGFP-positive cells with a spontaneously activating viral genome from the majority EGFP-negative cells and analysed their gene expression profiles by RNA-seq using Illumina HiSeq2500. Ingenuity pathway analysis on more than 2000 differentially expressed genes between the lytically infected (EGFP-positive) and latently infected (EGFP-negative) cell populations identified the biological pathways involved in the reactivation. Virus-reactivating cells exhibited differential expression of a significant number of viral genes, with hierarchical differences in expression levels. Downregulation of a number of host genes including those directly involved in T-cell activation, such as CD3, CD28, ICOS and phospholipase C, was also noticed in the LCL undergoing lytic switch.

## Abbreviations

BAC, bacterial artificial chromosome; CPM, count per million; DE, differentially expressed; d.p.i., days post infection; EGFP, enhanced green fluorescent protein; FACS, fluorescence-activated cell sorting; FDR, false discovery rate; GTF, general transfer format; HIP1, Huntingtin interacting protein 1; HSV, herpes simplex virus; IE, immediate early; ICOS, inducible T-cell co-stimulator; IPA, Ingenuity Pathway Analysis; LAT, latent associated transcript; LCL, lymphoblastoid cell line; LPL, lipoprotein lipase; MD, Marek’s disease; MDV, Marek’s disease virus; NGF, neuronal/nerve growth factor; RT-qPCR, quantitative reverse transcription PCR; Th, T-helper; US, unique short.

## Introduction

Marek’s disease virus (MDV) is a widely prevalent alphaherpesvirus of poultry, associated with Marek’s disease (MD) and characterized by rapid-onset CD4^+^TCRαβ^+^T-cell tumours at high incidence in susceptible hosts. Initial infection and subsequent spread of MDV occurs through the inhalation of infectious virus in dust. After a short lytic phase in B lymphocytes [~2–7 days post infection (d.p.i.)], MDV establishes a life-long latent infection in T-lymphocytes. Subsequently, the MDV lifecycle is completed by the virus replication in the feather follicle epithelium, the site from which infectious cell-free virus is shed into the environment through the dander [1, 2]. Neoplastic transformation of latently infected CD4^+^ lymphocytes in susceptible birds results in lymphomas. These tumours and the lymphoblastoid cell lines (LCLs) derived from them are mainly monoclonal with clonally restricted T cell receptor (TCR) profiles [3–5].

Latency is a distinct feature of herpesvirus infections, where the virus silently persists in the infected cell with occasional reactivation events leading to lytic infection. For example, in humans, all herpesviruses initially cause a primary infection before entering a latent state from which it can reactivate and cause recurrent lesions [6]. In herpes simplex virus type 1 (HSV-1) and HSV-2 [7, 8] infections, reactivation is triggered by various factors attributed to either the virus itself, host genetics or the surrounding environment. For example, the appearance of HSV cold sores results from a wide range of environmental stressors, including mental stress, fatigue, hormonal changes, dental surgery, cranial trauma, fever and exposure to UV light [9]. Understanding the mechanistic events during reactivation is therefore of great interest. Various *in vitro* culture systems to elucidate the pathways and molecular events involved in reactivation have indicated the roles of both host factors such as neuronal/nerve growth factor (NGF) [10–13] and viral factors such as latent associated transcripts (LATs), VP16, ICP0 [14–18].

Compared to tudies on human herpesviruses, little is known about the factors that regulate MDV latency and reactivation [19]. The potential involvement of epigenetic factors such as DNA methylation and histone modifications of the repeat regions of the viral genome in the maintenance of latency have been demonstrated [20]. It was also shown that MDV-1 telomeric repeats are essential for efficient integration, enhanced tumour formation and capacity for reactivation [21]. These results suggested the roles of multiple factors, both for efficient maintenance of the virus in the latent state and mobilization of the virus genome during reactivation. Studies on LCLs derived from tumours induced by recombinant MDV-expressing lacZ marker showed that the marker gene was expressed with the same kinetics as lytic viral genes pp38, US1, gB, gI, and US10 after treatment with 5′-iododeoxyuridine [22]. This study also showed that MDV-encoded oncoprotein Meq, although normally associated with transformation and latency, could be detected in cells expressing the marker and the lytic antigens. Indeed, Meq expression in lytic and latent/tumour cells was also demonstrated in other independent studies [23]. Other studies characterized the cell surface phenotypes of lytically infected cells and transformed cell lines. For example, MHC class II was upregulated during lytic infection on the surface of an MDV-derived cell line, RP1, upon bromodeoxyuridine-induced transcriptional activation [24]. This was considered a unique response to MDV and is thought to assist in enhancing cell-to-cell contact and spread of MDV to activated T-lymphocytes. On the other hand, downregulated surface expression of MHC Class I (BF) glycoproteins by blocking their transport to the cell surface during active, but not latent, infection of chicken cells has also been reported [25]. Taken together, these studies showed the involvement of both host and viral factors in the latency to lytic switch of MDV.

The work reported here aimed to investigate differential gene expression during spontaneous lytic switch of MDV within the microenvironment of the transformed cell line. Previous reports, including our preliminary work, demonstrated that cell lines such as MSB-1 contain 1–10 % of the cells displaying lytic phenotype, as demonstrated by expression of pp38 [26]. However, the mechanism and the microenvironment by which MDV is reactivated in the small proportion of cells in these cell lines remain unknown. The presence of these lytically infected subpopulations in the cell lines suggests a process of spontaneous reactivation, and these cells provide a unique opportunity to understand the pathways involved in MDV lytic switch.

In the present study, we established two LCLs from tumours induced by pRB1B-UL47eGFP virus [27] as a tool to study spontaneous lytic switch of MDV. We have recently used transmission electron microscopy to demonstrate morphogenesis of herpesvirus particles in the enhanced green fluorescent protein (EGFP)-expressing cells of one of these cell lines [28]. Global gene expression profiling using RNA-seq was used to examine the transcriptome changes associated with lytic switch. We present detailed analysis of the global changes in the host and viral transcriptome of cells undergoing spontaneous lytic switch of MDV from latency in these LCLs.

## Results

### Raw data

The raw sequencing data have been submitted to the European Nucleotide Archive under accession number PRJEB14979.

### Recombinant pRB1B-UL47eGFP MDV and generation of cell lines

**Fig. 1.**
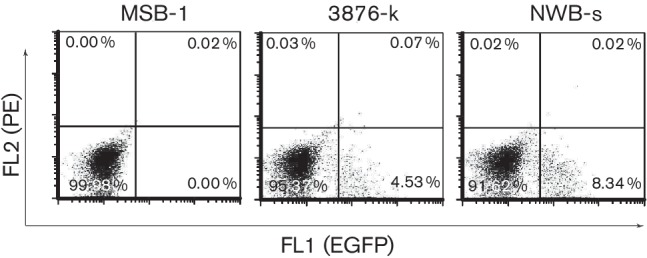
Use of recombinant fluorescent MDV virus to identify lytic switch in established MDV LCL. Two cell lines (3867-k and NWB-s) were generated from tumours derived from P line birds infected with pRB1B- UL47eGFP. Dot plot shows unstained MSB-1 cell line as a negative control; Unstained 3867-k cell line showing approximately 4.5 % cells positive for UL47eGFP expression and unstained NWB-s cell line showing approximately 8.3 % cells positive for UL47eGFP expression.

Inbred P line (MHC B^19/19^) white leghorn chickens infected with pRB1B-UL47eGFP [27] showed expression of EGFP in the feather follicle epithelium (data not shown) confirming its use as a potential marker for lytically infected cells. Two cell lines (NWB-s and 3867-k) were established from primary lymphomas from spleen and kidney of two separate birds respectively following serial passages of *ex vivo* tumour cell cultures. Flow cytometric analyses revealed that although majority of the cells in culture were EGFP^−^, the two cell lines demonstrated continued presence of a minor population of EGFP^+^ cells during culture (Fig. 1). Overall percentages of the EGFP^+^ cells were consistent with the numbers of pp38^+^ cell populations in reactivating LCLs previously reported [22, 29]. Compared to the multiple steps of fixation and permeabilization to demonstrate the virus-encoded lytic proteins in the cytoplasmic compartment of conventional MDV-transformed LCLs, NWB-s and 3867-k cell lines with the readily demonstrable EGFP marker were quicker for sorting of reactivating cells with minimal manipulation. Moreover these cell lines contained monoclonal CD4^+^ cells of Vβ1 TCR types (data not shown) a profile demonstrated for majority of MDV derived LCL [3]. Thus these two LCLs formed excellent samples for comparing changes in gene expression between latency and lytic switch in EGFP^−^ and EGFP^+^ populations respectively.

### RNA-seq of EGFP^+^ versus EGFP^−^ cell populations

Single-cell suspensions of the cultures for the two cell lines were used to purify EGFP^+^ and EGFP^−^ subpopulations using fluorescence-activated cell sorting (FACS). The purified cells were used to extract high quality RNA for subsequent sequencing. Total reads and the insert sizes from the subpopulations of cells were comparable (Table 1). Filtered sequencing reads were mapped to *Gallus gallus* (Ensembl release 74) and gallid herpesvirus 2 (MDV type 1, NC_002229) reference genome sequences. The total numbers and percentages of aligned reads for host and virus genomes were comparable between the two cell lines. The gene expression profiles in EGFP^−^ and EGFP^+^ populations were clearly separable in terms of biological coefficient of variation (data not shown). The proportion of host chicken genes that accounted for more than 99 % of the reads in the EGFP^−^ latent cell populations in both cell lines were reduced to 76 and 83 % in the EGFP^+^ activated population in the two cell lines. This change in the expression of host genes was accompanied by an increase in the proportion of reads of viral genes to 16.23 and 23.22 % in the EGFP^+^ NWB-s and 3867-k, respectively (Table 1), further confirming the lytic switch of the virus in these populations. We also examined the numbers of reads of EGFP transcripts in the latent and reactivated cell populations of the two cell lines. As expected, the two reactivated cell populations showed a 100–1000-fold increase in EGFP expression compared to the latent cells (Table 1).

**Table 1.** Summary of RNA-seq reads aligned to *Gallus gallus*, MDV and EGFP

LCL Identifier		Total reads	Insert size (nt)	Number of reads (%) aligned with		
				*Gallus gallus*	MDV	EGFP
NWB-s	EGFP^−^	155 401 762	167	128 254 562 (99.56 %)	556 004 (0.44 %)	1708 (0.001 %)
	EGFP^+^	130 052 456	178	79 874 012 (83.77 %)	15 470 241 (16.23 %)	126 931 (0.10 %)
3867-k	EGFP^−^	145 789 034	166	122 750 138 (99.78 %)	270 291 (0.22 %)	363 (0.0002 %)
	EGFP^+^	148 125 420	165	78 560 470 (76.78 %)	23 758 049 (23.22 %)	297 887 (0.20 %)

**Fig. 2.**
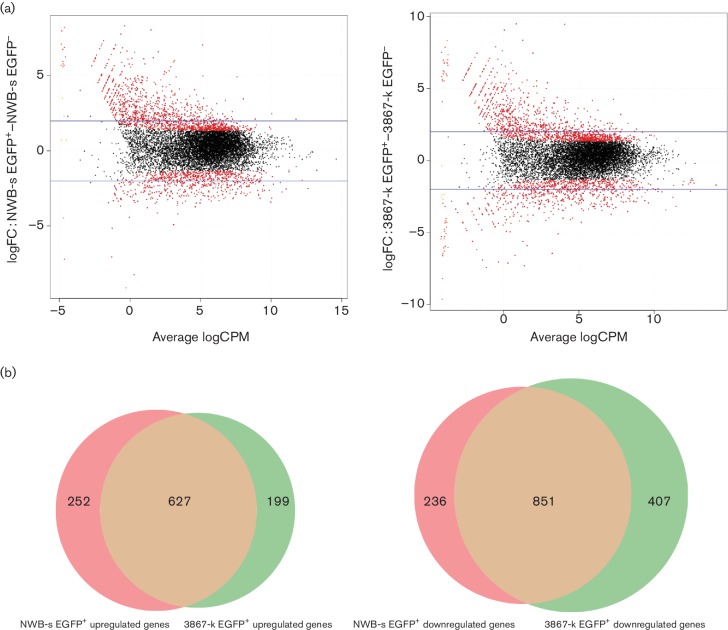
Analysis of DE genes. (a) The edgeR smear plot of log fold change (FC) against the average log count per million (CPM). (Left) DE genes in NWB-s EGFP^+^ versus NWB-s EGFP^−^. (Right) DE genes in 3867-k EGFP^+^ versus 3867-k EGFP^−^. Blue horizontal lines represent four fold changes. The FDR cut-off 0.05 was applied to display the significantly DE genes, which are highlighted in red. (b) Venn diagram representing the common upregulated and downregulated genes in the two LCLs.

Differentially expressed (DE) genes in positive and negative samples that are potentially associated with reactivation of MDV are also likely to be involved in a number of biological processes that play a role in immune evasion or modulation, transformation and virus lifecycle. We therefore aimed to identify all the DE genes to decipher their association with pathways and cellular networks. To this end, edgeR software was used to perform pair-wise tests separately between EGFP^−^ and EGFP^+^ from NWB-s and 3867-k cell line data. DE genes were further filtered at a false discovery rate (FDR) <0.05 (Fig. 2a).

**Fig. 3.**
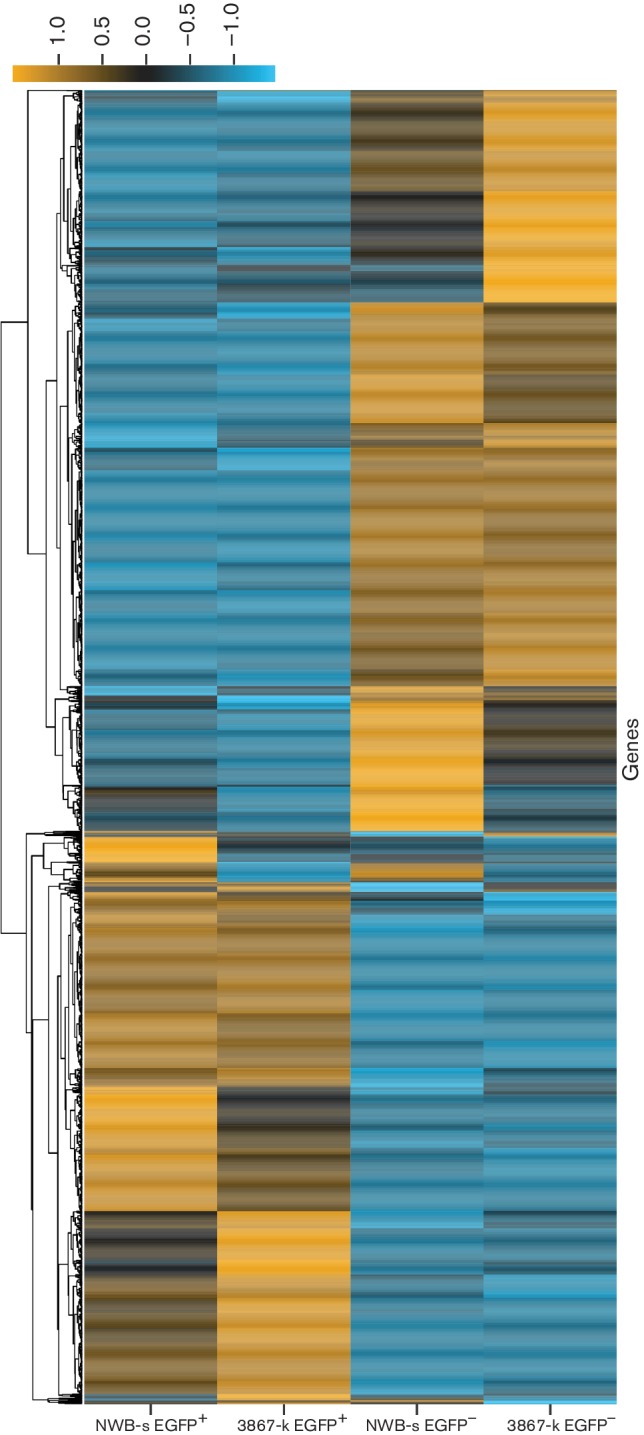
Heat map of DE host genes in latent and reactivated LCLs. Comparisons of the relative expression levels of the genes in EGFP^+^ populations of 3867-k and NWB-s LCLs compared with EGFP^−^ populations. The expression levels are highlighted in blue and yellow scale.

*DE host genes*. A high number of DE genes were observed between EGFP^+^ and EGFP^−^ samples in the two cell lines using 0.25 biological coefficient of variation. Interestingly, there were similarities in the profiles of DE genes in the latent and reactivated populations of the two cell lines. The 3867-k cell line showed a total of 2093 DE genes, of which 1262 were downregulated and 831 upregulated. The NWB-s cell line showed a total of 1975 DE genes of which 1092 were downregulated and 883 upregulated. The results also showed that 1478 DE genes, including 851 downregulated and 627 upregulated genes, were common in both lines (Fig. 2b). In order to reveal the relative expression pattern of these significant DE genes in the NWB-s and 3867-k cell lines, the count per million (CPM) of the normalized expression values of these genes in all four samples were displayed as heat maps where genes were clustered using correlation (Fig. 3). This analysis revealed a remarkable similarity in the expression profiles between the two cell lines. A list of the top 100 upregulated and downregulated genes in the EGFP^+^ subpopulation from each LCL, ranked by order of significance, is shown in Table S1(a–d) (available in the online Supplementary Material).

**Fig. 4.**
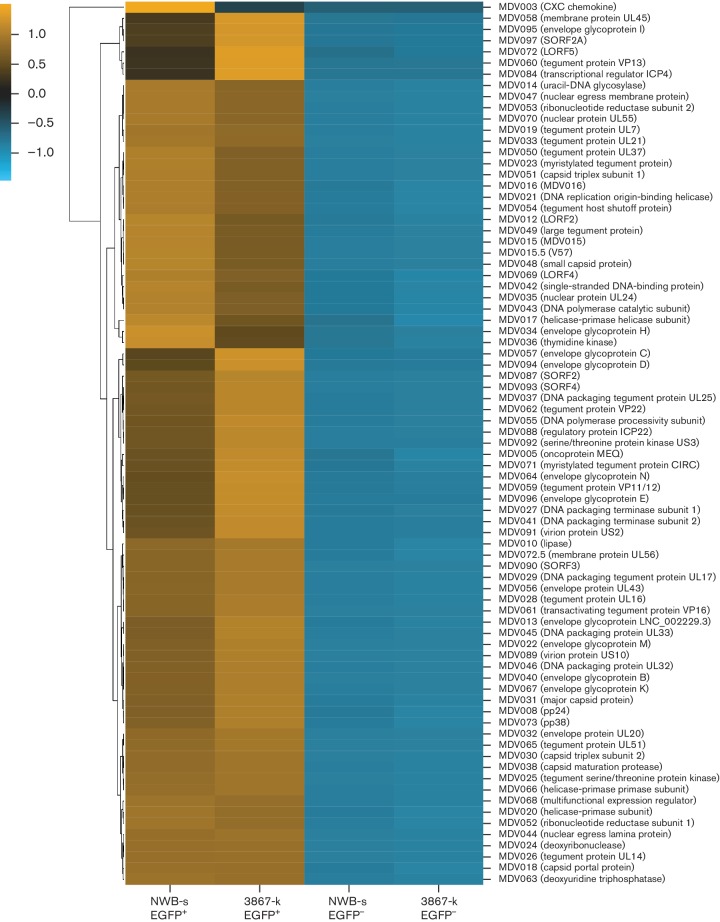
Heat map of DE viral genes in latent and reactivated LCLs. Comparisons of the relative expression levels of the genes in EGFP^+^ populations of 3867-k and NWB-s LCLs compared with EGFP^−^ populations. The expression levels are highlighted in blue and yellow scale.

*DE viral genes*. Analysis of the viral transcripts [30] from both cell lines revealed 82 DE genes, all of which were upregulated in the EGFP^+^ population of reactivating cells as opposed to the EGFP^−^ latent cell population. In order to determine the relative expression pattern of the significantly expressed viral genes in the NWB-s and 3867-k cell lines, normalized expression values of these genes in all four samples were displayed as heat maps where genes were clustered using correlation (Fig. 4). CPM values for viral transcript from both LCLs are presented in Table S2(a). The CPM values were also used to analyse relative changes in the levels of each transcript between the two populations. This analysis showed the remarkable similarities of the two cell lines in relation to the rankings of the transcripts based on highest and lowest fold changes (Table S2b). Amongst the transcripts that showed the highest relative fold changes were the transcriptional regulator ICP4, envelope glycoprotein E, protein SORF3, envelope glycoprotein I, virion protein US2 and tegument protein VP13. On the other hand, MDV-encoded proteins such as MEQ oncoprotein, LORF5, helicase-primase helicase subunit, myristylated tegument protein CIRC and protein LORF4 showed the least levels of changes in expression (Table S2b).

### Validation of gene array results

**Fig. 5.**
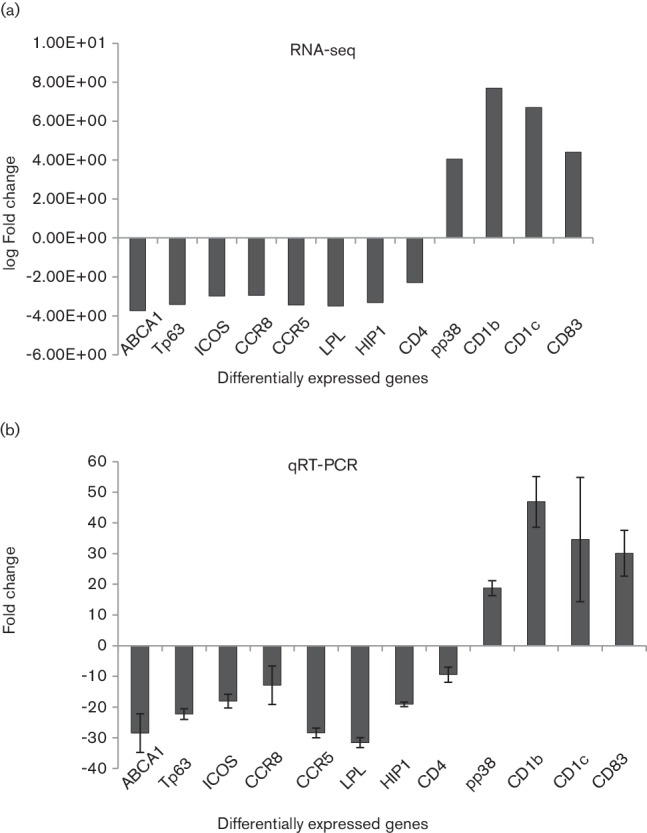
Expression of NWB-s LCL genes selected for validation. (a). Fold-change values obtained from RNA-seq data: ABCA1, Tp63, ICOS, putative CCR8, putative CCR5, LPL, HIP1, CD4 were downregulated, and CD1b, CD1c, CD83, pp38 genes were upregulated. (b) Expression was determined by RT-qPCR. Expression was analysed relative to the internal control (HMBS) in the EGFP^+^ cells relative to the EGFP^−^ cells and expressed as fold change (2^−ΔΔCT^). The expression pattern where ABCA1, Tp63, ICOS, CCR8, CCR5, LPL, HIP1, CD4 were downregulated and CD1b, CD1c,CD83, pp38 genes were upregulated is similar to that obtained by RNA-seq. Error bars indicate standard deviation (sd).

Graphical representation of fold-change values obtained from RNA-seq data for 12 selected genes is shown in Fig. 5(a). This analysis indicated that ATP-binding cassette transporter (ABCA1), Tp63, inducible T-cell co-stimulator (ICOS), putative CCR8, putative CCR5, lipoprotein lipase (LPL), Huntingtin interacting protein 1 (HIP1), CD4 transcripts were downregulated in the EGFP^+^ cells relative to the EGFP^−^ cells. Moreover, CD1b, CD1c and CD83 transcripts were upregulated in the EGFP^+^ cells relative to the EGFP^−^ cells. Furthermore, pp38, an MDV viral transcript expressed during lytic infection, was also upregulated in the EGFP^+^ cells. Expression changes of transcripts selected from the downregulated and upregulated genes were analysed by using the SYBR-green RT-qPCR method. Analysis of expression of each of these genes relative to the internal control (HMBS transcript) in the EGFP^+^ cells relative to EGFP^−^ cells indicated that ABCA1, Tp63, ICOS, putative CCR8, putative CCR5, LPL, HIP1 CD4 were downregulated while CD1b, CD1c, CD83, pp38 transcripts were upregulated (Fig. 5b). This observation was consistent with data obtained by RNA-seq with all selected genes comparable in the direction and relative magnitude from these two independent expression analysis platforms, thus validating the data generated by the RNA-seq method.

### Pathway analysis

**Fig. 6.**
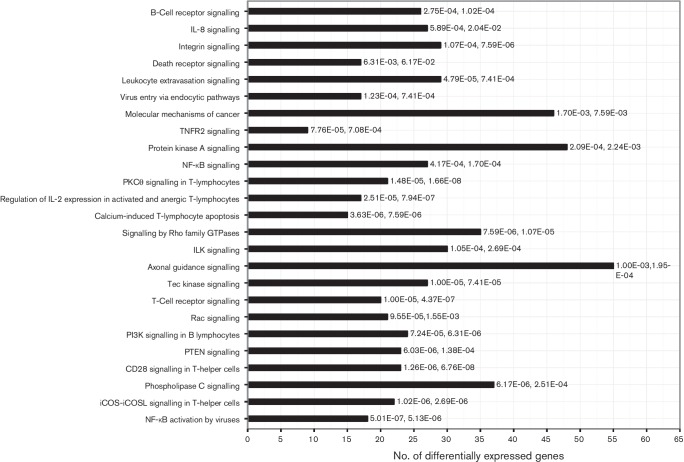
Top canonical pathways of DE genes in EGFP^+^ cells from NWB-s and 3867-k LCLs. All DE genes were submitted as a group. *P-*values indicating the significance of each pathway are shown starting with those for NWB-s followed by those for 3867-k.

In order to investigate biological functions of DE genes, accession numbers of genes of interest were imported into Ingenuity Pathway Analysis (IPA) along with their corresponding expression *P*-values and log fold-change values. This enabled comparison of over-represented signalling and metabolic pathways and gene functions for EGFP^+^ and EGFP^−^ samples. The top recognized pathways of upregulated and downregulated genes represented in both EGFP^+^ samples are shown in Fig. 6. Since the samples were derived from MDV-transformed T-cell lines, genes associated with immune evasion/modulation, viral infection and tumour were expected to be DE in this data set. Indeed, the top significantly DE genes were those involved in immunological disease, infectious disease, inflammatory response and metabolic disease. Moreover, they were associated with molecular functions such as cellular development, cell-to-cell signalling and interaction, cell morphology, cellular function and maintenance, and cellular movement.

## Discussion

MD is a major disease of poultry with huge economic and animal welfare significance, and it is also an excellent natural biomedical model for rapid-onset T-cell lymphomas. Recent research on this disease has led to major breakthroughs in our understanding of the basic transforming attributes of MDV such as the role of virus-encoded oncoprotein Meq and oncomicroRNA miR-M4 [23, 31]. Moreover, the availability of infectious bacterial artificial chromosome (BAC) clones of MDV allowing the generation of recombinant viruses expressing fluorescent marker genes has now enabled studies looking into the role of viral gene products in replication and pathogenesis. At the same time, recent advances in the chicken genome annotation and associated bioinformatics tools have enabled more in-depth analysis of gene expression profiles in MDV-infected cells. Data generated from such studies are beginning to provide insights into the complex MDV–host interactions.

The work presented here aimed to analyse the changes in global transcription during the switch from latency to lytic infection in an MDV-transformed cell line model. This is of major interest because MDV, similar to other herpesviruses, is an obligate intracellular parasite, dependent on the host cell machinery for replication during active lytic infection. Latency and reactivation are indeed hallmarks of persistent infections seen in all herpesviruses, where the viral lifecycle involves both silent and productive infection without rapidly damaging the host cells [32]. Reactivation, a process which in most cases is associated with disease symptoms and complications, may be provoked by a combination of external and/or internal cellular stimuli [6].

In MD tumour cells and LCLs, it is increasingly becoming clear that the latency is maintained through epigenetic mechanisms that include methylation and chromatin modifications [19, 20, 33]. In order to gain insights into the lytic switch process in MDV-transformed LCLs, two cell lines, 3867-k and NWB-s, were generated from kidney and spleen tumours, respectively, induced in P line birds infected with pRB1B-UL47eGFP, a recombinant virus expressing EGFP marker as a fusion protein with UL47 [27]. As the viral genomes are maintained in a latent state, EGFP expression is shut off in both cell lines. However, we found that a small proportion of cells spontaneously expressed EGFP at levels similar to those of MDV lytic genes such as pp38 seen in cells with reactivating virus [22, 29]. We have also recently shown that these cells produce virus particles [28]. The ability to isolate these spontaneously reactivating fluorescent cells presented an ideal resource to analyse, with minimal manipulation, cell populations showing viral reactivation in relation to the majority latent cell population.

This analysis generated in excess of 2000 DE genes, consistent with the extensive intracellular, biochemical and morphologic changes that accompany the transition from the latency phase to the reactivated state. However, there is limited information regarding the role of most of these gene products and the biological/molecular pathways during MDV infection. Nevertheless, by inferring to the known roles in the relatively well-studied mammalian system, and based on historical anecdotal evidence, we selected a few candidates from the significantly highly expressed gene list for exploring the possible roles for their differential expression within the context of active viral infection. Our data revealed that changes in the host transcription in the reactivating population involved downregulation of key cellular pathways such as TCR, co-stimulatory molecules and factors in associated signalling systems. This suggested that MDV reactivation resulted in inhibition of the common T-cell activation pathways such as TCR, ICOS and CD28. TCR ligation initiates a central signalling pathway critical for regulating T-cell biology. It is therefore not surprising that MDV, like many other lymphotropic viruses, have evolved mechanisms to modulate TCR signalling. In this regard, it has been documented that T-lymphotropic viruses such as human T-lymphotropic virus type 1, herpesvirus saimiri and human herpesvirus 6 can all modulate the TCR function [34–37]. However, direct interference of the TCR pathways by MDV has not yet been demonstrated. Nevertheless, it is known that MDV preferentially infects activated T-cells [19] and therefore one could speculate that the virus may have yet-unidentified mechanisms for modulating the TCR pathways and related molecular interactions. Given the central role of the PI3-kinase signalling pathway during reactivation and the shared role of kinases during immune receptor signalling pathways, it is not surprising that reactivation in an immune cell would inevitably involve manipulation of the key immune receptor signalling pathway. Consistent with this view, a number of genes, including ICOS, CD4, lymphocyte-specific protein tyrosine kinase (LCK), zeta-chain (TCR)-associated protein kinase 70 kDa (ZAP70), IL2-inducible T-cell kinase (ITK), phospholipase C, gamma 1 (PLCy1), IL-2R and CD3 chains, were downregulated following reactivation. This transcription profile indicated a scenario where genes either directly or indirectly involved in TCR signalling are downmodulated. IPA analyses showed that ICOS is mainly involved in iCOS-iCOSL signalling in T-helper (Th) cells, primary immunodeficiency signalling and the Th cell differentiation pathway. Moreover, ICOS has many upstream regulators such as IL2, TNF, TCF3, miR-29b, TCR, IL15, CSF2, CD3, ionomycin, miR-66, cyclosporine A, STAT3, CD28, IL4 and NFκB, among others. Due to these interactions, it is involved in several functions concerned with cell death and survival, morphology, signalling, cell-mediated immune response, tumour morphology and cancer, immune cell trafficking, and inflammatory response. Furthermore, ZAP70 was also downregulated and IPA analysis indicated that it may be associated with many pathways such as ICOS signalling in Th, CD28 signalling, calcium-induced T-lymphocyte apoptosis, phospholipase C-signalling, T-cell receptor signalling and PKC0 signalling in T-lymphocytes. These pathways constitute an intricate web of interactions that are essential during T-cell activation and downregulation of some or in this case the majority of these gene products and would inevitably inhibit activation of the affected T-cells.

In contrast, some genes, such as those involved in immune regulation, were found to be upregulated. For example, MHC-II transcripts were upregulated in the reactivated cells. Indeed, upregulation of MHC-II was previously reported in primary MDV-infected cell populations [24], and was hypothesized to be one possible way that the strictly cell-associated MDV virus is propagated into new target cells resulting in enhanced MHC–TCR interaction. Similarly, upregulation of other immune system modulating/regulating genes (such as CD83) was observed. CD83 is mainly involved in crosstalk between dendritic cells and natural killer cells, dendritic cell maturation and TREM1 signalling. In addition, CD83 has many upstream regulators such as IL2, TNF, TGFB1, lipopolysaccharide, dexamethasone, IL15, CD40L6, CSF2, NR3CL, CD3, STAT3 and NFκB, hence its involvement in functions of cellular development, growth and proliferation. Furthermore, soluble CD83 has immunosuppressive roles such as the inhibition of T-cell stimulation [38], which would presumably affect the functional ability of the immune cells and benefit viral replication. We speculated that it is this inhibitory role that might be of relevance to its upregulation in cell-bearing actively replicating virus. Interestingly, in addition to the protein coding genes, we also found that gga-miR-142 (ENSGALG00000018344) is in the top 100 downregulated genes in the activated EGFP^+^ cell population in both NWB-s and 3867-k cell lines (Table S1b, d respectively). We have previously reported that gga-miR-142 is highly expressed in MDV-transformed LCLs such as MSB-1 [39], and its downregulation following lytic switch in both cell lines could be functionally relevant. Based on the gene expression profiles and pathway analysis, it appears that the final outcome of the reactivation process in MDV-transformed LCLs is to dampen the immune responsiveness and activation status of the T-cells. Such a process may provide a direct benefit to the virus by rendering the actively infected host cell unable to control viral replication.

Our studies also showed that other metabolic pathways such as those involved in the lipid metabolism (ABCA1, ABCG1, ABCG4) were also affected in the reactivating cell population. IPA showed that ABCA1 is involved in three major pathways: LXR/RXR activation, PPARalpha/RXRalpha activation and LPS/IL1 mediated inhibition of RXR function, which in turn leads to multifunctional pathways. Although the involvement of these metabolic pathways has not been previously reported in MD, impairment of these pathways is known to be associated with HIV infectivity [40] and increased risk of atherosclerotic plaque formation and coronary artery disease. Interestingly, a phenomenon referred to ‘foamy’ cell development in arterial lesions has also been observed following MDV infection [41]. Moreover, infection of cultured chicken arterial smooth muscle cells with MDV led to significant alterations in cholesterol metabolism which contributed to the 7–10-fold increase in free and esterified cholesterol accumulation in these cells, partly due to decreased excretion of free cholesterol [42]. Based on past reports and the current data, we would hypothesize that cellular lipid metabolism and membrane structures such as lipid rafts may be modified to enable viral assembly, envelopment and egress during reactivation and active MDV replication.

As expected, we also observed the differential expression of multiple viral genes in the reactivating cell populations from both cell lines (Fig. 4). During herpesvirus reactivation, multiple interactions between the virus gene products and the host cellular pathways do occur. These host–viral interactions occur at various levels; probably starting with the primary role in initiation of reactivation process itself and the subsequent secondary role in maintenance of reactivated state mainly concerned with regulation of active viral replication during lytic infection including ensuring a permissive cellular environment. Within the well-studied human HSV reactivation, only a handful of viral proteins have been implicated in initiation of reactivation including immediate early (IE) proteins ICPO, ICP4 and others such as viral thymidine kinase and tegument protein VP16 [6, 18, 43].

In our global transcriptome analysis study described here it is rather difficult to distinguish the role played by individual viral products for various outcomes. This would require further tailored studies that employ overexpression or knockout/knockin studies of potential candidate genes in a similar cellular environment. However, it is evident from the list of DE viral genes that a pattern of upregulation was observed with some viral transcripts that encode proteins with a reported reactivation role in other herpesviruses and hence the potential significance of their upregulation can be inferred. These included a clear upregulation of transcripts for products with reported reactivation activities in HSV systems such as ICP4 and VP16, with ICP4 ranked second and fourth top upregulated transcript in the NWB-s and 3867-k cell lines, respectively. Thymidine kinase was also moderately upregulated in both cell lines.

Although the absolute requirement for ICP4 and viral thymidine kinase in HSV reactivation remains unclear and lack consensus from multiple independent studies, the involvement of VP16 in reactivation is well documented [17]. VP16 is classified as a late gene protein but previous studies demonstrated that it is expressed *de novo* (unaffected by normal modulation of late gene expression) as a pre-IE gene in neurons following reactivation [44]. Functionally, VP16 is able to transactivate the five IE HSV genes and initiate the viral lytic cycle. The mechanism of action involves interaction with host-cell proteins including host-cell factor-1 (HCF-1), a cell-cycle regulator and octomer binding protein-1 (Oct-1), a POU domain transcription factor, to form the VP16-induced complex that binds to TAATGARAT elements within the five HSV-1 IE gene promoters [17]. Even more significant is the report that viral mutants that lack the transactivation function of VP16 do not exit the latent state *in vivo* and have no detectable viral proteins during latency [44]. For this reason, VP16 is a potential candidate for anti-HSV therapy. Although the kinetics of expression of most MDV genes is not well studied, upregulation of VP16 among the other viral transcripts in the EGFP^+^ cell population in our RNA-seq data would suggest a possible role in the MDV reactivation process that is likely to involve a similar mechanism to that in HSV.

Also notable was upregulation of various genes encoding for viral proteins involved in virus assembly, such as envelope glycoprotein E, I, D, B, M K and various tegument proteins such as tegument protein VP13, UL14, Vp22, UL16, UL51. Also on this list of upregulated genes are glycoprotein C and US2, previously reported to be associated with the horizontal transmission of the virus [45]. Moreover, it was also noted that the expression of viral genes was not random across the whole genome. Viral genes located in the unique short (US) regions appeared to be overrepresented among the most highly upregulated genes (with all DE US transcripts ranked among the top 15 positions in each cell line). The significance of this pattern of expression is yet to be determined.

In summary, we demonstrated differential expression of multiple host and viral genes in the lytic switch process within two independent MD-derived LCLs as indicated by RNA-seq data. Although linking the DE genes to direct or indirect host–virus interaction events, and hence deciphering the mechanistic basis of lytic switch in MDV, is beyond the scope of this study, the data could nonetheless aid in identifying potential host candidate genes and/or pathways that are prime targets of modulation by the virus during the process of lytic switch. Taking into consideration the fact that MDV is a model transforming herpesvirus and looking at the examples of selected DE genes described above, the data could provide insights into several interesting areas. Firstly, this data may provide useful indicators of the pathways involved in manipulation of host cellular mechanisms by the virus to maintain latency and sustain the tumour microenvironment, such as modulation of the host cell proliferation. Secondly, this data may also provide useful indicators regarding modulation of the host response in cells harbouring reactivated virus, possibly to escape presentation and or recognition by the host immune system and the viral genes involved. It is conceivable that regulation of latency and lytic switch observed *in vitro* in these two LCLs may be different to *in vivo* events. Nonetheless, deciphering such differences in these tumour cell lines would greatly contribute to our current understanding of host–virus interaction in herpesvirus biology, especially as the mechanisms and the determinants of the relationship between MDV latency and transformation remain unclear. However, the work reported here reveals a list of possible candidate genes that could be crucial in future studies aimed at defining MDV latency.

## Methods

### Experimental infection

Inbred P line (MHC, B^19/19^) specific pathogen-free (SPF) white leghorn chickens were maintained at the Poultry Production Unit of the Pirbright Institute. One-day-old birds were infected with 1000 p.f.u. dose of pRB1B-UL47eGFP virus [27] by intra-abdominal injection as described previously [46, 47]. All experiments were carried out under specific project licences issued by the UK Home Office.

### Tumour cell preparations, cell culture and maintenance of LCLs

Tumours were aseptically removed from birds at post mortem examination and lymphocytes prepared by Histopaque-1083 density-gradient centrifugation and LCLs established using methods described previously [3]. LCLs were adapted to grow at 38.5 °C in 5 % CO_2_ in RPMI 1640 medium containing 10 % foetal bovine serum (FBS), 10 % tryptose phosphate broth and 1 % sodium pyruvate. Methods of the maintenance of MSB-1 have been described previously [48].

### Flow cytometry and sorting

Single-cell suspensions of cultured LCL were washed twice with PBS containing 5 % FBS with centrifugation at 450 ***g*** for 5 min at room temperature. The cell pellets were suspended in cold PBS/5 % FBS containing 1 µM DAPI for subsequent exclusion of dead cells. EGFP^+^ and EGFP^−^ subpopulations were sorted by FACS using FACSAria II (BD bioscience) to a purity of over 93 %. Cells were sorted at five different passage levels (21–25 for NWB-S and 48–52 for 3867-k cell lines) for each subpopulation. To maintain relative uniformity during sorting, aliquots of cells were removed from culture in batches of a maximum 1×10^7^ cells at a time and immediately sorted.

### RNA isolation

Cells were disrupted by suspension in RLT buffer (QIAGEN) and stored at −80 °C. RNA was prepared from pooled cells sorted at the five different passage levels for each subpopulation to a total of four pooled RNA samples. RNA was extracted with the RNeasy Mini kit (QIAGEN) according to the manufacturer’s instructions. Contaminating DNA was digested with RNase-free DNase 1 (QIAGEN) on a column for 15 min at room temperature. RNA was eluted with 50 µl RNase-free water (QIAGEN) and stored at −80 °C.

### cDNA library preparation and RNA-seq

Total RNA was processed to make cDNA libraries using TruSeq Technology according to the manufacturer’s instructions (Illumina, San Diego, CA) and four libraries were subsequently sequenced by using Illumina HiSeq2500.

### Data filtering, mapping reads and transcriptome analysis

In order to filter the data, adaptor sequences were removed and poor quality reads (lower than Q20) were discarded using cutadapt v1.4.1 with default parameters. Non-redundant reads were then mapped to the *Gallus gallus* and gallid herpesvirus 2 (MDV type 1) reference genome sequences with TopHat-2.0 using default settings [49]. The *Gallus gallus* reference genome sequence and gene structure information (GTF, general transfer format) were downloaded from the ensembl database (release 74). The MDV genome sequence and annotation were fetched from the NCBI FTP database (accession id: NC_002229). HTseq-count of HTSeq-0.5.3 [50] was then used to sum the read counts for each gene to generate expression levels. Moreover, CPM values were obtained and used to normalize the counts and the edgeR statistical package and an over-dispersed Poisson model were used to determine significant differences in gene counts across negative and positive samples to account for variability in gene expression [51]. DE genes filtered at FDR <0.05 were then mapped to known pathways/gene ontology terms and their biological functions determined. Data were analysed by using IPA [52] by importing gene accession numbers along with the corresponding expression *P*-values and log fold-change values. The IPA ‘core analysis’ function was used to interpret data in the context of biological functions, pathways and gene regulation networks.

### RT-qPCR validation of gene expression

RT-qPCR analysis was performed on an ABI Model 7500 Real-Time instrument (Applied Biosystems, Foster City, CA, USA) using the Verso 1-step SYBR Green RT-qPCR kit plus Rox system (Thermo scientific, Waltham, MA, USA) according to the manufacturer’s instructions. Briefly, RNA (12.5 ng) was mixed with 6.5 µl of 2×1 Step qPCR mix, 0.5 µl enhancer, 0.1 µl enzyme and 1 µl of mixed forward and reverse primers at 1 µM working concentration, in a 13 µl final reaction volume. RT-qPCR conditions were as follows: one cycle, 50 °C for 30 min, 95 °C for 15 min followed by 40 cycles of 95 °C for 15 s, 60 °C for 30 s and 72 °C for 30 s. Gene changes were determined using the 2^−ΔΔ^ ^Ct^ method [53] by normalizing to hydroxymethylbilane synthase expression, which did not vary significantly in RNA prepared from EGFP^+^ and EGFP^−^ cells. Primer sequences and further details regarding the primers are provided in Table 2.

**Table 2.** Primer sequences for qRT-PCR

Gene	Type	Sequence	Amplicon size (bp)
ABCA1	Forward	5′-CCTCTGTTTATCTTCTTCATCCTGATC-3′	106
	Reverse	5′-AATGTTCCTGCTGAGGGCATA-3′	
Tp63	Forward	5′-CCTCTCCATGCCTTCAACGT-3′	114
	Reverse	5′-CGTGAAATAATCCACACAGGATGA-3′	
ICOS	Forward	5′-ACACTGCTGATTCTTATTCCTTAAGTGA-3′	163
	Reverse	5′-TACATTGCCACTTGAAAGAAACCTA-3′	
CCR8	Forward	5′-AACTGACTGCCTTGTGGGTTTATAT-3′	202
	Reverse	5′-TGGAAATCCGCCAGCAA-3′	
CCR5	Forward	5′-CGGTTTAGCGTTACTCTTGATGTAATAA-3′	85
	Reverse	5′-TGGACGTGTTCAGCTGATGAC-3′	
LPL	Forward	5′-GACCAAGGTAGACCAGCCATTC-3′	136
	Reverse	5′-CACCAATGTCCACTTCTGTGTAGAT-3′	
HIP1	Forward	5′-ACCGTCAAGCTGCTCTTCAAAC-3′	114
	Reverse	5′-TGGAGCGATAGAAGAGGTCCTT-3′	
CD4	Forward	5′-GAGTGGCCCAGCAGGGATA-3′	113
	Reverse	5′-CAGTTAAATCACTCTGGGCAGTACA-3′	
CD1b	Forward	5′-TTGCAGCCTGTGTGGATCTG-3′	66
	Reverse	5′-TGCATGAGATGACTGCAAAGG-3′	
CD1c	Forward	5′-AGCAGGCTGGTGCAGATGTA-3′	121
	Reverse	5′-TGGCCCTCGTAAGCAATGTC-3′	
CD83	Forward	5′-CACCCTGTGCAATGTTTGGA-3′	73
	Reverse	5′-CAAAGCATGTCACAGCAACATCT-3′	
pp38	Forward	5′- GAAAACAGAAGCGGAATGCG-3′	69
	Reverse	5′-CGATCCAAAGCGCTCATCTC-3′	
HMBS	Forward	5′-GGTTGAGATGCTCCGTGAGTTT-3′	153
	Reverse	5′-GGCTCTTCTCCCCAATCTTAGAA-3′	

## Supplementary Data

520 Supplementary File 1
