# Supplementary material for: Differentially expressed genes during spontaneous lytic switch of Marek's disease virus in lymphoblastoid cell lines determined by global gene expression profiling
Source: J Gen Virol. 2017 May 5;98(4):779–90. doi: 10.1099/jgv.0.000744 (PMC5657026; doi:10.1099/jgv.0.000744)
Supplement: Supplementary File 1 [file jgv-98-779-s001.pdf]

Supplementary Table S1a. Top 100 Upregulated genes in NWB-s EGFP$^+$

Ensembl ID	Gene name
ENSGALG00000027865	CD1.2
ENSGALG00000012494	CD1A1
ENSGALG00000019831	*
ENSGALG00000020999	DYNLL1
ENSGALG00000010171	NFKBIE
ENSGALG00000000974	IGSF1
ENSGALG00000019033	RELT
ENSGALG00000028495	*
ENSGALG00000014436	GPAT2
ENSGALG00000028505	PSMD4
ENSGALG00000009452	GALNT16
ENSGALG00000000182	*
ENSGALG00000023828	LDHD
ENSGALG00000000452	TVB
ENSGALG00000008537	EPHB3
ENSGALG00000026152	*
ENSGALG00000003932	SDC4
ENSGALG00000009470	SERPINI1
ENSGALG00000024507	gga-mir-22
ENSGALG00000023768	EPHA2
ENSGALG00000026291	CDH3
ENSGALG00000014555	C12ORF57
ENSGALG00000000287	*
ENSGALG00000026909	U8
ENSGALG00000010129	PLK3
ENSGALG00000005802	FLT4
ENSGALG00000014725	PLK2
ENSGALG00000002840	GRM4
ENSGALG00000002436	*
ENSGALG00000005680	PSD
ENSGALG00000029057	VSX1
ENSGALG00000008546	FGD5
ENSGALG00000009902	WDR65
ENSGALG00000027133	CD83
ENSGALG00000026166	VDR
ENSGALG00000027415	GRIN2C
ENSGALG00000002442	ANKS4B
ENSGALG00000001262	CLIC4
ENSGALG00000010071	MAB21L2
ENSGALG00000002051	DNASE1
ENSGALG00000026396	BG1
ENSGALG00000002467	MTSS1L
ENSGALG00000013370	SEMA3F
ENSGALG00000004645	*
ENSGALG00000011476	Sep-11
ENSGALG00000000585	MYL4
ENSGALG00000005631	TRPM2
ENSGALG00000013922	ZP1
ENSGALG00000000420	KLHDC7A
ENSGALG00000007501	P2RX3
ENSGALG00000002678	CSPG4
ENSGALG00000008995	MAMDC4
ENSGALG00000006565	*
ENSGALG00000013457	PLEKHO1
ENSGALG00000019797	PTH3R
ENSGALG00000028482	CCDC65
ENSGALG00000007420	
ENSGALG00000000136	BLEC2
ENSGALG00000004476	SEMA3G
ENSGALG00000006099	ZFPM1
ENSGALG00000005924	MGAT4B
ENSGALG00000009719	PCDH10
ENSGALG00000015152	BOC
ENSGALG00000007480	AXIN1
ENSGALG00000015404	*
ENSGALG00000003022	*
ENSGALG00000028044	ADMP
ENSGALG00000013587	COL2A1
ENSGALG00000027526	COL9A2
ENSGALG00000003595	SARM1
ENSGALG00000021198	HAUS3
ENSGALG00000001545	RAB40B
ENSGALG00000000785	HYAL1
ENSGALG00000009340	TRIM63
ENSGALG00000004297	DNAH1
ENSGALG00000021855	CCDC39
ENSGALG00000016608	ADCY3
ENSGALG00000004320	FAT2
ENSGALG00000007349	RASL12
ENSGALG00000025718	*
ENSGALG00000016087	FBXO41
ENSGALG00000005298	ST6GALNAC6
ENSGALG00000027288	*
ENSGALG00000011212	IL23R
ENSGALG00000023364	CDC42EP4
ENSGALG00000001111	DNAH9
ENSGALG00000023707	*
ENSGALG00000000458	TMPRSS9
ENSGALG00000002759	PEAK1
ENSGALG00000001035	*
ENSGALG00000001132	APBB3
ENSGALG00000010435	RASGRP3
ENSGALG00000008215	FRMD5
ENSGALG00000000722	*
ENSGALG00000006835	TNNC2
ENSGALG00000013034	CECR2
ENSGALG00000003123	CNTNAP1
ENSGALG00000012610	CTSL2
ENSGALG00000010126	BEST4
ENSGALG00000026802	*

*Uncharacterised genes

Supplementary Table S1b. Top 100 downregulated genes in NWB-s EGFP⁺

Ensembl ID	Gene name
ENSGALG00000026566	CDC42SE1
ENSGALG00000028938	*
ENSGALG00000004635	*
ENSGALG00000026335	ARPC2
ENSGALG00000003868	*
ENSGALG00000018344	gga-mir-142
ENSGALG00000013546	GZMK
ENSGALG00000009046	CFTII
ENSGALG00000014496	PROM1
ENSGALG00000002939	*
ENSGALG00000009628	KCTD12
ENSGALG00000015433	ABCA1
ENSGALG00000005754	*
ENSGALG00000014750	*
ENSGALG00000026225	PSMD12
ENSGALG00000016173	UBASH3A
ENSGALG00000008047	*
ENSGALG00000014910	COLEC12
ENSGALG00000028411	*
ENSGALG00000001161	FLI1
ENSGALG00000014455	LPAR5
ENSGALG00000016687	P2RY8
ENSGALG00000004252	*
ENSGALG00000026574	POUV
ENSGALG00000002786	PSTPIP1
ENSGALG00000015425	LPL
ENSGALG00000007874	IL18
ENSGALG00000004842	NT5M
ENSGALG00000011732	CCR5
ENSGALG00000013442	*
ENSGALG00000002213	*
ENSGALG00000027834	*
ENSGALG00000007324	TP63
ENSGALG00000017331	GDPD5
ENSGALG00000019060	MMP27
ENSGALG00000021355	PTPRZ1
ENSGALG00000015755	ZNF704
ENSGALG00000006278	*
ENSGALG00000027747	*
ENSGALG00000005868	RAP1GAP2
ENSGALG00000007546	*
ENSGALG00000000930	HIP1
ENSGALG00000006637	*
ENSGALG00000027334	*
ENSGALG00000023787	*
ENSGALG00000006344	GSTT1
ENSGALG00000015679	SAMSN1
ENSGALG00000025991	GLOD5
ENSGALG00000001023	*
ENSGALG00000024118	SLAMF1
ENSGALG00000028580	gga-mir-147
ENSGALG00000002712	LIPG
ENSGALG00000013548	GZMA
ENSGALG00000014754	TRBC2
ENSGALG00000011447	GADL1
ENSGALG00000025926	MZB1
ENSGALG00000007838	NLRC3
ENSGALG00000014179	SBK1
ENSGALG00000009718	FAM53B
ENSGALG00000007846	*
ENSGALG00000016362	SH3YL1
ENSGALG00000001410	SPNS3
ENSGALG00000012525	FMNL2
ENSGALG00000003857	ITK
ENSGALG00000011733	CCR2
ENSGALG00000011081	*
ENSGALG00000013372	IL7R
ENSGALG00000012545	CYTIP
ENSGALG00000002329	CCL1
ENSGALG00000001486	ZAP70
ENSGALG00000023909	ENTPD2
ENSGALG00000012420	LGALS1
ENSGALG00000019061	*
ENSGALG00000005192	CD5
ENSGALG00000001226	ABCA2
ENSGALG00000001004	*
ENSGALG00000005539	ITM2C
ENSGALG00000000600	PTPN7
ENSGALG00000007878	SDHD
ENSGALG00000006707	NOX1
ENSGALG00000014456	ACRBP
ENSGALG00000001571	MYO1F
ENSGALG00000011955	CX3CR1
ENSGALG00000023439	DFNB59
ENSGALG00000014561	PTPN6
ENSGALG00000013941	PDE7B
ENSGALG00000001505	NGEF
ENSGALG00000002234	UNC13D
ENSGALG00000007418	CD3D
ENSGALG00000008312	GSAP
ENSGALG00000020575	ERMN
ENSGALG00000017068	KL
ENSGALG00000014508	CD38
ENSGALG00000006516	MTHFS
ENSGALG00000020615	STK32C
ENSGALG00000008656	ICOS
ENSGALG00000019751	MICAL1
ENSGALG00000021405	*
ENSGALG00000015448	TOX
ENSGALG00000002192	PTPRC

*Uncharacterised genes

Supplementary Table S1c. Top 100 upregulated genes in 3867-k $EGFP^{+}$

Ensembl ID	Gene name
ENSGALG00000012494	CD1A1
ENSGALG00000010171	NFKBIE
ENSGALG00000027865	CD1.2
ENSGALG00000028505	PSMD4
ENSGALG00000000974	IGSF1
ENSGALG00000027362	NFS1
ENSGALG00000000182	*
ENSGALG00000019831	*
ENSGALG00000000287	*
ENSGALG00000005802	FLT4
ENSGALG00000026291	CDH3
ENSGALG00000026152	*
ENSGALG00000027133	CD83
ENSGALG00000014436	GPAT2
ENSGALG00000024507	gga-mir-22
ENSGALG00000006647	DUSP8
ENSGALG00000026909	U8
ENSGALG00000004521	GPX3
ENSGALG00000002051	DNASE1
ENSGALG00000010126	BEST4
ENSGALG00000002442	ANKS4B
ENSGALG00000020999	DYNLL1
ENSGALG00000004297	DNAH1
ENSGALG00000001111	DNAH9
ENSGALG00000006447	PHLDA2
ENSGALG00000008546	FGD5
ENSGALG00000010071	MAB21L2
ENSGALG00000015820	CA13
ENSGALG00000005680	PSD
ENSGALG00000007480	AXIN1
ENSGALG00000003782	PAX-7
ENSGALG00000006236	TPH1
ENSGALG00000005631	TRPM2
ENSGALG00000019033	RELT
ENSGALG00000009470	SERPINI1
ENSGALG00000002436	*
ENSGALG00000003595	SARM1
ENSGALG00000003420	ZBTB8A
ENSGALG00000007349	RASL12
ENSGALG00000013587	COL2A1
ENSGALG00000026396	BG1
ENSGALG00000001262	CLIC4
ENSGALG00000028482	CCDC65
ENSGALG00000008537	EPHB3
ENSGALG00000010129	PLK3
ENSGALG00000015152	BOC
ENSGALG00000009909	PRPH2
ENSGALG00000018237	gga-let-7i
ENSGALG00000014555	C12ORF57
ENSGALG00000016087	FBXO41
ENSGALG00000000420	KLHDC7A
ENSGALG00000023818	*
ENSGALG00000000997	GJC1
ENSGALG00000023768	EPHA2
ENSGALG00000023828	LDHD
ENSGALG00000023707	*
ENSGALG00000002678	CSPG4
ENSGALG00000000452	TVB
ENSGALG00000029057	VSX1
ENSGALG00000007420	*
ENSGALG00000028578	PIP5K1A
ENSGALG00000013370	SEMA3F
ENSGALG00000000585	MYL4
ENSGALG00000007211	CDH22
ENSGALG00000000902	C1orf172
ENSGALG00000012610	CTSL2
ENSGALG00000021559	CD14
ENSGALG00000002840	GRM4
ENSGALG00000000136	BLEC2
ENSGALG00000019797	PTH3R
ENSGALG00000004645	
ENSGALG00000007669	EGR1
ENSGALG00000026773	gga-mir-222a
ENSGALG00000027288	
ENSGALG00000005077	F8
ENSGALG00000006099	ZFPM1
ENSGALG00000010382	C3H6ORF154
ENSGALG00000011902	TAGLN
ENSGALG00000003022	*
ENSGALG00000005031	DRP2
ENSGALG00000004501	*
ENSGALG00000013221	ARHGEF33
ENSGALG00000013861	TNFAIP3
ENSGALG00000013922	ZP1
ENSGALG00000000293	*
ENSGALG00000027400	*
ENSGALG00000000497	*
ENSGALG00000007335	KBTBD13
ENSGALG00000003868	*
ENSGALG00000013457	PLEKHO1
ENSGALG00000028016	*
ENSGALG00000018266	gga-let-7a-1
ENSGALG00000028616	CELSR2
ENSGALG00000028975	FRMPD3
ENSGALG00000028269	*
ENSGALG00000005141	HMCN1
ENSGALG00000028044	ADMP
ENSGALG00000006835	TNNC2
ENSGALG00000006501	GGT5
ENSGALG00000005210	WNK2

*Uncharacterised genes

Supplementary Table S1d. Top 100 downregulated genes in 3867-k EGFP$^+$

Ensembl ID	Gene name
ENSGALG00000022679	WPKCI
ENSGALG00000000386	*
ENSGALG00000022674	HINTW
ENSGALG00000022683	HINTW
ENSGALG00000022685	*
ENSGALG00000005785	*
ENSGALG00000001756	ATP5A1W
ENSGALG00000026335	ARPC2
ENSGALG00000022174	*
ENSGALG00000009628	KCTD12
ENSGALG00000024075	IL10R1
ENSGALG00000007846	*
ENSGALG00000011733	CCR2
ENSGALG00000006014	PRKCB
ENSGALG00000014910	COLEC12
ENSGALG00000004481	EDA
ENSGALG00000026858	BTG2
ENSGALG00000002939	*
ENSGALG00000001226	ABCA2
ENSGALG00000013442	*
ENSGALG00000028635	AIF1L
ENSGALG00000007511	ITGB2
ENSGALG00000021439	*
ENSGALG00000004635	*
ENSGALG00000026970	*
ENSGALG00000015425	LPL
ENSGALG00000026574	POUV
ENSGALG00000023876	PRKCQ
ENSGALG00000006344	GSTT1
ENSGALG00000027170	*
ENSGALG00000015433	ABCA1
ENSGALG00000015755	ZNF704
ENSGALG00000002329	CCL1
ENSGALG00000009453	*
ENSGALG00000011732	CCR5
ENSGALG00000021355	PTPRZ1
ENSGALG00000026588	*
ENSGALG00000007874	IL18
ENSGALG00000029006	SYPL1
ENSGALG00000018344	gga-mir-142
ENSGALG00000011081	
ENSGALG00000006360	H2AFY
ENSGALG00000000519	UBXN11
ENSGALG00000005065	PLA2G4A
ENSGALG00000005754	*
ENSGALG00000017331	GDPD5
ENSGALG00000003700	PSMD12
ENSGALG00000004252	*
ENSGALG00000013546	GZMK
ENSGALG00000019060	MMP27
ENSGALG00000014455	LPAR5
ENSGALG00000007546	*
ENSGALG00000009289	SLC18A2
ENSGALG00000028272	*
ENSGALG00000012420	LGALS1
ENSGALG00000003437	ADAM8
ENSGALG00000013941	PDE7B
ENSGALG00000000395	BTF3
ENSGALG00000009046	CFTII
ENSGALG00000001486	ZAP70
ENSGALG00000002786	PSTPIP1
ENSGALG00000027747	*
ENSGALG00000007878	SDHD
ENSGALG00000008047	*
ENSGALG00000016687	P2RY8
ENSGALG00000010333	CNOT6L
ENSGALG00000028495	*
ENSGALG00000008312	GSAP
ENSGALG00000014844	NKAIN2
ENSGALG00000023787	*
ENSGALG00000015691	SMC2
ENSGALG00000007303	LEPREL1
ENSGALG00000016173	UBASH3A
ENSGALG00000001161	FLI1
ENSGALG00000002712	LIPG
ENSGALG00000025991	GLOD5
ENSGALG00000016128	B3GALT5
ENSGALG00000000600	PTPN7
ENSGALG00000007324	TP63
ENSGALG00000014944	GCNT4
ENSGALG00000000930	HIP1
ENSGALG00000013372	IL7R
ENSGALG00000016442	RRM2
ENSGALG00000027751	*
ENSGALG00000001410	SPNS3
ENSGALG00000009227	*
ENSGALG00000008656	ICOS
ENSGALG00000003426	RHBDL3
ENSGALG00000014750	*
ENSGALG00000014561	PTPN6
ENSGALG00000014456	ACRBP
ENSGALG00000014508	CD38
ENSGALG00000008323	COL17A1
ENSGALG00000019751	MICAL1
ENSGALG00000015747	ZBTB10
ENSGALG00000028580	gga-mir-147
ENSGALG00000009718	FAM53B
ENSGALG00000002234	UNC13D
ENSGALG00000008725	KIF16B
ENSGALG00000006278	*

*Uncharacterised genes

Supplementary Table S2a. Differentially expressed MDV genes following spontenous lytic switch in LCLs.

(Gene list displayed by rank order)

ORF	Gene Identity	NWB-s EGFP^{+}	3867-k EGFP+	NWB-s EGFP-	3867-k EGFP-
MDV003	CXC chemokine	2492.929	274.2314	0.043075	0
MDV084	transcriptional regulator ICP4	2612.007	5463.77	6.202831	6.724245
MDV096	envelope glycoprotein E	9944.006	14801.01	58.5823	16.22393
MDV090	protein SORF3	913.6341	1039.15	5.449015	1.128229
MDV095	envelope glycoprotein I	3354.127	6066.889	21.8176	5.189854
MDV092	serine/threonine protein kinase US3	732.0212	1008.472	5.922842	1.444133
MDV091	virion protein US2	401.8212	569.5388	3.316792	0.78976
MDV089	virion protein US10	4141.847	4987.296	36.98007	10.33458
MDV060	tegument protein VP13	5723.617	11587.35	51.49642	16.53984
MDV065	tegument protein UL51	651.5388	704.7015	5.793617	1.714908
MDV048	small capsid protein	1816.635	1403.164	16.41166	3.835979
MDV094	envelope glycoprotein D	670.4709	1060.307	6.202831	1.489262
MDV088	regulatory protein ICP22	2763.238	3787.92	28.12812	6.543728
MDV093	protein SORF4	292.2796	396.6184	2.929115	0.496421
MDV087	protein SORF2	624.9552	822.3145	6.719734	1.647214
MDV040	envelope glycoprotein B	3708.576	4402.323	42.10602	8.348895
MDV022	envelope glycoprotein M	897.8808	1084.665	10.14421	2.053377
MDV026	tegument protein UL14	917.7412	923.5442	12.27644	2.008248
MDV058	membrane protein UL45	105.2094	196.3291	1.335332	0.496421
MDV041	DNA packaging terminase subunit 2	536.8775	766.9258	7.408937	2.098506
MDV032	envelope protein UL20	193.2309	209.9728	2.627588	0.609244
MDV061	transactivating tegument protein VP16	7453.215	8602.811	109.3464	21.61687
MDV051	capsid triplex subunit 1	889.4134	745.3887	12.92257	2.662621
MDV023	myristylated tegument protein	485.1448	405.5696	7.193561	1.263617
MDV044	nuclear egress lamina protein	2728.046	2782.649	41.43836	9.251478
MDV067	envelope glycoprotein K	1288.956	1535.316	19.83614	4.467787
MDV059	tegument protein VP11/12	4866.554	7242.748	76.41544	13.58388
MDV047	nuclear egress membrane protein	1173.338	1048.644	18.52234	3.993931
MDV015.5	protein V57	798.9445	617.9836	12.85795	2.933395
MDV097	protein SORF2A	337.5703	600.1898	5.49209	2.188764
MDV024	deoxyribonuclease	4849.423	4886.365	81.32601	13.80952
MDV033	tegument protein UL21	394.3947	361.3291	6.612046	1.263617
MDV025	tegument serine/threonine protein kinase	2153.445	2256.021	37.38929	6.092437
MDV062	tegument protein VP22	3122.525	4213.101	55.22243	9.612511
MDV057	envelope glycoprotein C	1616.709	2628.553	28.90347	7.130407
MDV050	tegument protein UL37	500.4199	415.2802	8.851957	1.714908
MDV030	capsid triplex subunit 2	3382.033	3594.303	61.23142	14.82493
MDV015	MDV015	1621.547	1268.436	29.7219	6.99502
MDV037	DNA packaging tegument protein UL25	420.7533	565.877	8.184291	1.195923
MDV014	uracil-DNA glycosylase	1698.429	1504.286	34.09403	7.24323
MDV045	DNA packaging protein UL33	306.8795	387.613	6.224369	0.992842
MDV046	DNA packaging protein UL32	3580.974	4275.596	74.41244	13.35823
MDV019	tegument protein UL7	601.3534	558.499	12.4272	1.760037
MDV055	DNA polymerase processivity subunit	3064.913	4251.292	68.42498	10.35714
MDV038	capsid maturation protease	1329.38	1411.6	29.78651	5.212418
MDV028	tegument protein UL16	106.2221	122.9566	2.261449	0.29334
MDV064	envelope glycoprotein N	178.2934	269.2947	3.898307	1.33131
MDV068	multifunctional expression regulator	4875.922	4709.348	112.6202	21.12045
MDV029	DNA packaging tegument protein UL17	157.3922	179.8915	3.618318	0.78976
MDV027	DNA packaging terminase subunit 1	748.0839	1088.788	17.74699	2.820573
MDV063	deoxyuridine triphosphatase	5413.418	5337.884	130.2595	30.16884
MDV053	ribonucleotide reductase subunit 2	2930.476	2615.397	71.89254	17.57781
MDV049	large tegument protein	2770.496	2148.689	68.70497	13.80952
MDV070	nuclear protein UL55	3069.471	2742.124	76.35082	16.44958
MDV066	helicase-primase primase subunit	1463.002	1528.047	36.44163	7.062714
MDV056	envelope protein UL43	5333.695	6084.303	141.8682	25.6108
MDV052	ribonucleotide reductase subunit 1	2771.087	2622.477	82.27366	18.4127
MDV012	protein LORF2	4698.697	3630.027	153.1539	34.04995
MDV054	tegument host shutoff protein	599.0185	511.6003	20.63303	3.362123
MDV013	envelope glycoprotein LNC_002229.3	307.6672	385.4972	10.96264	3.316993
MDV021	DNA replication origin-binding helicase	704.6218	598.8878	25.41438	3.520075
MDV031	major capsid protein	3784.276	4562.766	139.2406	30.21397
MDV018	capsid portal protein	583.3778	579.8462	22.82987	2.798008
MDV034	envelope glycoprotein H	3120.303	2003.68	126.3181	18.88655
MDV020	helicase-primase subunit	1481.315	1411.627	60.86528	10.33458
MDV016	MDV016	495.7783	416.9077	21.10686	4.061625
MDV035	nuclear protein UL24	171.6545	139.1229	8.442743	0.880019
MDV072.5	membrane protein UL56	5104.598	5776.817	259.8512	87.37006
MDV010	lipase	32668.31	36157.51	1664.125	554.4794
MDV036	thymidine kinase	762.2338	508.2097	40.18918	4.783691
MDV073	protein pp38	8541.514	10182.18	467.8615	138.2532
MDV042	single-stranded DNA-binding protein	4322.897	3400.036	245.2487	56.70479
MDV043	DNA polymerase catalytic subunit	1111.703	897.7214	63.23442	13.92235
MDV008	pp24	4404.111	5286.672	269.6293	73.08668
MDV069	protein LORF4	1107.456	923.3543	77.0831	18.63834
MDV071	myristylated tegument protein CIRC	764.7093	1092.938	65.23742	24.07641
MDV005	oncoprotein MEQ	1.547197	8.110306	0.064613	0.180517
MDV017	helicase-primase helicase subunit	403.1433	297.8029	41.00761	6.99502
MDV072	protein LORF5	1379.256	2781.51	157.0522	55.89247

Supplementary Table S2b. Differentially expressed MDV genes following spontenous lytic switch in LCLs.

(Gene list displayed by rank order)

NWB-s					3867-k				
**Rank**	**ORF**	**Location**	**Description**	**EGFP+/ EGFP-**	**Rank**	**ORF**	**Location**	**Description**	**EGFP+/ EGFP-**
1	MDV003	TRL	CXC chemokine					Not ranked	
2	MDV084	IRS	transcriptional regulator ICP4	421.1	2	MDV095	US	envelope glycoprotein I	1169.0
3	MDV096	US	envelope glycoprotein E	169.7	3	MDV090	US	protein SORF3	921.0
4	MDV090	US	protein SORF3	167.7	4	MDV096	US	envelope glycoprotein E	912.3
5	MDV095	US	envelope glycoprotein I	153.7	5	MDV084	IRS	transcriptional regulator ICP4	812.5
6	MDV092	US	serine/threonine protein kinase US3	123.6	6	MDV093	US	protein SORF4	799.0
7	MDV091	US	virion protein US2	121.1	7	MDV091	US	virion protein US2	721.2
8	MDV089	US	virion protein US10	112.0	8	MDV094	US	envelope glycoprotein D	712.0
9	MDV060	UL	tegument protein VP13	111.1	9	MDV060	UL	tegument protein VP13	700.6
10	MDV065	UL	tegument protein UL51	112.5	10	MDV092	US	serine/threonine protein kinase US3	698.3
11	MDV048	UL	small capsid protein	110.7	11	MDV088	US	regulatory protein ICP22	578.9
12	MDV094	US	envelope glycoprotein D	108.1	12	MDV059	UL	tegument protein VP11/12	533.2
13	MDV088	US	regulatory protein ICP22	98.2	13	MDV022	UL	envelope glycoprotein M	528.2
14	MDV093	US	protein SORF4	99.8	14	MDV040	UL	envelope glycoprotein B	527.3
15	MDV087	IRS/US	protein SORF2	93.0	15	MDV087	IRS/US	protein SORF2	499.2
16	MDV040	UL	envelope glycoprotein B	88.1	16	MDV089	US	virion protein US10	482.6
17	MDV022	UL	envelope glycoprotein M	88.5	17	MDV037	UL	DNA packaging tegument protein UL25	473.2
18	MDV026	UL	tegument protein UL14	74.8	18	MDV026	UL	tegument protein UL14	459.9
19	MDV058	UL	membrane protein UL45	78.8	19	MDV062	UL	tegument protein VP22	438.3
20	MDV041	UL	DNA packaging terminase subunit 2	72.5	20	MDV028	UL	tegument protein UL16	419.2
21	MDV032	UL	envelope protein UL20	73.5	21	MDV065	UL	tegument protein UL51	410.9
22	MDV061	UL	transactivating tegument protein VP16	68.2	22	MDV055	UL	DNA polymerase processivity subunit	410.5
23	MDV051	UL	capsid triplex subunit 1	68.8	23	MDV061	UL	transactivating tegument protein VP16	398.0
24	MDV023	UL	myristylated tegument protein	67.4	24	MDV058	UL	membrane protein UL45	395.5
25	MDV044	UL	nuclear egress lamina protein	65.8	25	MDV045	UL	DNA packaging protein UL33	390.4
26	MDV067	UL	envelope glycoprotein K	65.0	26	MDV027	UL	DNA packaging terminase subunit 1	386.0
27	MDV059	UL	tegument protein VP11/12	63.7	27	MDV025	UL	tegument serine/threonine protein kinase	370.3
28	MDV047	UL	nuclear egress membrane protein	63.3	28	MDV057	UL	envelope glycoprotein C	368.6
29	MDV015.5	UL	protein V57	62.1	29	MDV048	UL	small capsid protein	365.8
30	MDV097	US/TRS	protein SORF2A	61.5	30	MDV041	UL	DNA packaging terminase subunit 2	365.5
31	MDV024	UL	deoxyribonuclease	59.6	31	MDV024	UL	deoxyribonuclease	353.8
32	MDV033	UL	tegument protein UL21	59.6	32	MDV032	UL	envelope protein UL20	344.6
33	MDV025	UL	tegument serine/threonine protein kinase	57.6	33	MDV067	UL	envelope glycoprotein K	343.6
34	MDV062	UL	tegument protein VP22	56.5	34	MDV023	UL	myristylated tegument protein	321.0
35	MDV057	UL	envelope glycoprotein C	55.9	35	MDV046	UL	DNA packaging protein UL32	320.1
36	MDV050	UL	tegument protein UL37	56.5	36	MDV019	UL	tegument protein UL7	317.3
37	MDV030	UL	capsid triplex subunit 2	55.2	37	MDV044	UL	nuclear egress lamina protein	300.8
38	MDV015	UL	MDV015	54.6	38	MDV033	UL	tegument protein UL21	285.9
39	MDV037	UL	DNA packaging tegument protein UL25	51.4	39	MDV051	UL	capsid triplex subunit 1	279.9
40	MDV014	UL	uracil-DNA glycosylase	49.8	40	MDV097	US/TRS	protein SORF2A	274.2
41	MDV045	UL	DNA packaging protein UL33	49.3	41	MDV038	UL	capsid maturation protease	270.8
42	MDV046	UL	DNA packaging protein UL32	48.1	42	MDV047	UL	nuclear egress membrane protein	262.6
43	MDV019	UL	tegument protein UL7	48.4	43	MDV030	UL	capsid triplex subunit 2	242.4
44	MDV055	UL	DNA polymerase processivity subunit	44.8	44	MDV050	UL	tegument protein UL37	242.2
45	MDV038	UL	capsid maturation protease	44.6	45	MDV056	UL	envelope protein UL43	237.6
46	MDV028	UL	tegument protein UL16	47.0	46	MDV029	UL	DNA packaging tegument protein UL17	227.8
47	MDV064	UL	envelope glycoprotein N	45.7	47	MDV068	UL	multifunctional expression regulator	223.0
48	MDV068	UL	multifunctional expression regulator	43.3	48	MDV066	UL	helicase-primase primase subunit	216.4
49	MDV029	UL	DNA packaging tegument protein UL17	43.5	49	MDV015.5	UL	protein V57	210.7
50	MDV027	UL	DNA packaging terminase subunit 1	42.2	50	MDV014	UL	uracil-DNA glycosylase	207.7
51	MDV063	UL	deoxyuridine triphosphatase	41.6	51	MDV018	UL	capsid portal protein	207.2
52	MDV053	UL	ribonucleotide reductase subunit 2	40.8	52	MDV064	UL	envelope glycoprotein N	202.3
53	MDV049	UL	large tegument protein	40.3	53	MDV015	UL	MDV015	181.3
54	MDV070	UL	nuclear protein UL55	40.2	54	MDV063	UL	deoxyuridine triphosphatase	176.9
55	MDV066	UL	helicase-primase primase subunit	40.1	55	MDV021	UL	DNA replication origin-binding helicase	170.1
56	MDV056	UL	envelope protein UL43	37.6	56	MDV070	UL	nuclear protein UL55	166.7
57	MDV052	UL	ribonucleotide reductase subunit 1	33.7	57	MDV035	UL	nuclear protein UL24	158.1
58	MDV012	UL	protein LORF2	30.7	58	MDV049	UL	large tegument protein	155.6
59	MDV054	UL	tegument host shutoff protein	29.0	59	MDV054	UL	tegument host shutoff protein	152.2
60	MDV013	UL	envelope glycoprotein LNC_002229.3	28.1	60	MDV031	UL	major capsid protein	151.0
61	MDV021	UL	DNA replication origin-binding helicase	27.7	61	MDV053	UL	ribonucleotide reductase subunit 2	148.8
62	MDV031	UL	major capsid protein	27.2	62	MDV052	UL	ribonucleotide reductase subunit 1	142.4
63	MDV018	UL	capsid portal protein	25.6	63	MDV020	UL	helicase-primase subunit	136.6
64	MDV034	UL	envelope glycoprotein H	24.7	64	MDV013	UL	envelope glycoprotein LNC_002229.3	116.2
65	MDV020	UL	helicase-primase subunit	24.3	65	MDV078	IRL	CxC chemokine	107.6
66	MDV016	UL	MDV016	23.5	66	MDV012	UL	protein LORF2	106.6
67	MDV035	UL	nuclear protein UL24	20.3	67	MDV036	UL	thymidine kinase	106.2
68	MDV072.5	UL	membrane protein UL56	19.6	68	MDV034	UL	envelope glycoprotein H	106.1
69	MDV010	UL	lipase	19.6	69	MDV016	UL	MDV016	102.6
70	MDV036	UL	thymidine kinase	19.0	70	MDV073	UL/IRL	protein pp38	73.6
71	MDV073	UL/IRL	protein pp38	18.3	71	MDV008	TRL/UL	pp24	72.3
72	MDV042	UL	single-stranded DNA-binding protein	17.6	72	MDV072.5	UL	membrane protein UL56	66.1
73	MDV043	UL	DNA polymerase catalytic subunit	17.6	73	MDV010	UL	lipase	65.2
74	MDV008	TRL/UL	pp24	16.3	74	MDV043	UL	DNA polymerase catalytic subunit	64.5
75	MDV069	UL	protein LORF4	14.4	75	MDV042	UL	single-stranded DNA-binding protein	60.0
76	MDV071	UL	myristylated tegument protein CIRC	11.7	76	MDV072	UL	protein LORF5	49.8
77	MDV005	TRL	oncoprotein MEQ	23.9	77	MDV069	UL	protein LORF4	49.5
78	MDV017	UL	helicase-primase helicase subunit	9.8	78	MDV071	UL	myristylated tegument protein CIRC	45.4
79	MDV072	UL	protein LORF5	8.8	79	MDV005	TRL	oncoprotein MEQ	44.9
					80	MDV017	UL	helicase-primase helicase subunit	42.6

Boxed text indicate genes that were either ranked in same position or within 5 places in each cell line.
